# Different Vortex Vein Anomalies Observed in a Single Case: Macular Vortex Vein in One Eye and Varix of Vortex Vein Ampulla in the Other Eye

**DOI:** 10.7759/cureus.63668

**Published:** 2024-07-02

**Authors:** Keisuke Nitta, Hideo Akiyama

**Affiliations:** 1 Department of Ophthalmology, Gunma University Graduate School of Medicine, Maebashi, JPN

**Keywords:** macular vortex vein, enface optical coherence tomography, vortex vein, varix of vortex vein ampulla, ultra-widefield optical coherence tomography, posterior vortex vein, optical coherence tomography, multimodal imaging, choroidal vasculature

## Abstract

We present a 56-year-old female with a macular vortex vein in her right eye and a varix of vortex vein ampulla in the inferior nasal fundus of her left eye. The choroidal lesions were evaluated by multimodal imaging including fundoscopy with contact lens, ultra-widefield fundus photography, swept-souse optical coherence tomography (SS-OCT), enface image of widefield optical coherence tomography (widefield enface-OCT), and ultra-widefield fundus angiography. Widefield enface-OCT revealed submacular large choroidal vessels in the right eye. Ultra-widefield indocyanine green fluorescence angiography (UWICGA) of the right eye showed the dye of those submacular choroidal vessels drained from the ampullae beneath the macula. Fundoscopy revealed an elevated lesion with crescent shadows in the inferior nasal fundus of the left eye. Dynamic fundoscopy with compression of the left eye resulted in a diminishing of the elevation and release of the compression resulted in an enlargement of the elevation. Ultra-widefield fundus photography of the left eye in the right inferior gaze revealed an elevated lesion with a crescent shadow in the inferior nasal fundus, while it is not prominent in the primary gaze. The B-scan of SS-OCT revealed a hyporeflective lesion in the choroid beneath the elevated lesion of the left eye. The elevated lesion was consistent with the vortex vein ampulla on UWICGA. This is the first case where two different choroidal vascular anomalies, macular vortex vein and varix of vortex ampulla, coexist in a single patient. Multimodal imaging is useful to visualize and diagnose choroidal vascular anomalies.

## Introduction

The macular vortex vein and varix of vortex vein ampulla are both anomalies of choroidal vessels. The posterior vortex vein is a condition in which the vortex vein drainage is seen in the posterior pole [1]. The posterior vortex vein is often associated with high myopia and posterior staphyloma and is classified into five types according to the site of exit from the eye [1]. The type that the drainage is near the macula is called the macular vortex vein [2]. Varix of vortex vein ampulla is usually a benign, asymptomatic elevated mass, which is ill-defined and dark red in color [3]. The clinical importance of varix of vortex ampulla is that it could be a differential diagnosis of chorioretinal mass lesions, including choroidal melanoma, metastases to the choroid, subretinal hemorrhage, and choroidal hemangioma [4]. Although the common clinical perception of the number of vortex vein ampulla is 4 per eye, a recent report using ultra-widefield indocyanine green fluorescence angiography (UWICGA) revealed that the mean number of vortex vein ampullae in healthy human eyes is eight [5]. Multimodal imaging including UWICGA makes it possible to visualize a wide area of the choroid, which was previously difficult, and will lead to a better understanding of choroidal diseases. In this report, we present a case in which a macular vortex vein was found in one eye and a vortex vein ampulla in the other eye using multimodal imaging.

## Case presentation

**Figure 1:**
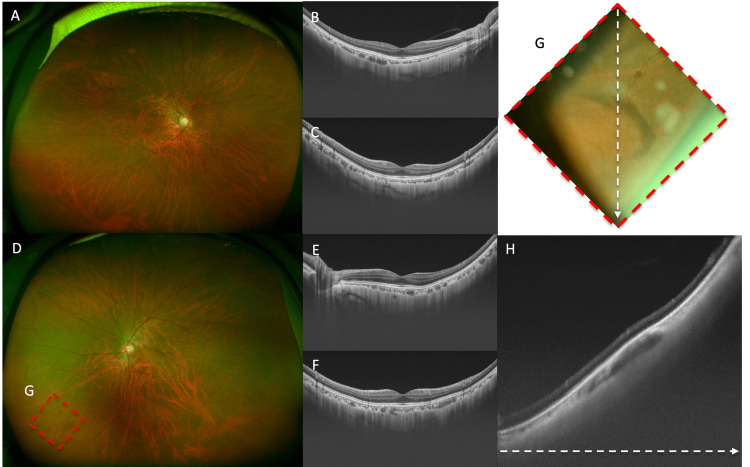
Ultra-widefield fundus color photographs and swept-souse optical coherence tomography (SS-OCT) images of both eyes, slit-lamp fundoscopy of elevated lesion in the left eye. The right eye showed no obvious abnormality on the ultra-widefield fundus color photographs (A) and no obvious abnormality including a posterior staphyloma in the posterior pole both in the horizontal (B) and vertical (C) B-scan slice of swept-souse optical coherence tomography (SS-OCT). Ultra-widefield fundus color photograph of the left eye (D) showed no obvious abnormality in the posterior pole; however, an elevated lesion and retinal hemorrhage were observed in the area outlined in (G). SS-OCT showed a posterior vitreous detachment (PVD) acertained by horizontal (E) and vertical (F) B-scan slices with the absence of a posterior hyaloid membrane. Observation of the area outlined by the red dotted line (G) on the inferior nasal fundus using a slit-lamp microscope and contact lens revealed a slightly elevated retinochoroidal mass. The vertical B-scan slice of SS-OCT in the same area (H), as shown by the white dotted line, revealed a hyporeflective lesion in the choroid.

A 56-year-old woman visited a local ophthalmologist with a chief complaint of floater in her left eye. An elevated lesion in the inferior peripheral fundus in her left eye was seen, and she was referred to our department with suspicion of a retinochoroidal tumor. At the initial visit, her corrected visual acuity was 20/16 in both eyes, with a myopia of -5.5 D in the right eye and -4.75 D in the left eye. Intraocular pressure was within normal range. No abnormalities in the anterior segment were observed in both eyes. No obvious abnormalities were found in the right fundus, including posterior staphyloma, as shown by ultra-widefield color fundus photograph (Optos®200Tx; Optos PLC, Dunfermline, United Kingdom) and swept-souse optical coherence tomography (SS-OCT) (DRI OCT-1 Triton; Topcon Corp., Tokyo, Japan) images of the posterior pole (Figure 1A-1C).

**Figure 2:**
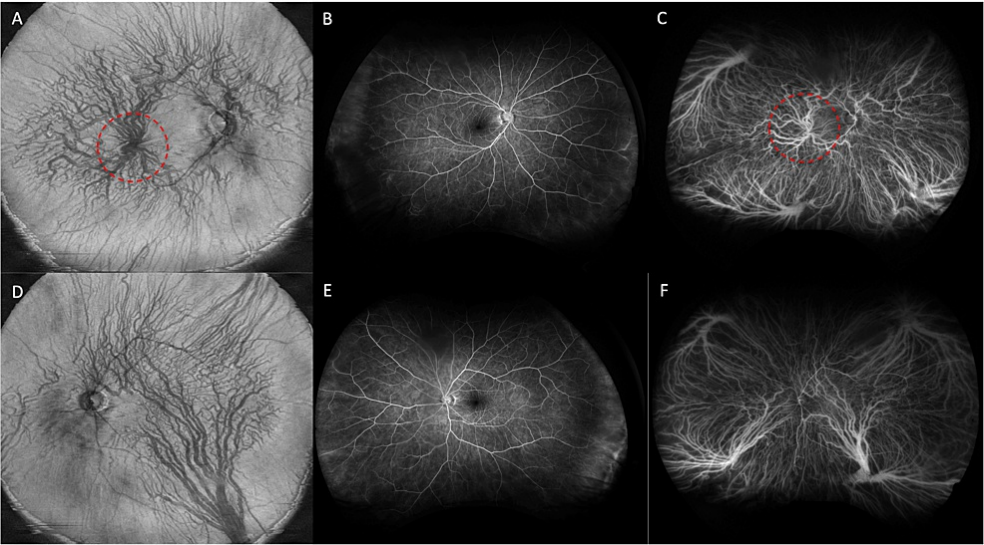
Ultra-widefield images of enface SS-OCT, ultra-widefield fluorescein angiography (UWFA), and ultra-widefield indocyanine green fluorescence angiography (UWICGA) of both eyes. In the ultra-widefield enface wept-souse optical coherence tomography (SS-OCT) image of the right eye (A), the vortex vein ampulla circled by the red dotted line is clearly seen in the posterior pole. In the ultra-widefield fluorescein angiography (UWFA) (two min 36 seconds) (B), no retinal vascular abnormality is seen in the posterior pole, while in the ultra-widefield indocyanine green fluorescence angiography (UWICGA) (two minutes 00 sec)(C), the inflow of the vortex vein into the posterior pole and the posterior vortex vein ampulla is seen. The left eye showed no obvious abnormal findings on the ultra-widefield enface SS-OCT (D), UWFA (one min 30 seconds) (E), or UWICGA (one minute 23 seconds) (F) at the primary gaze position.

Meanwhile, an elevated lesion was observed in the inferior nasal fundus of her left eye, as shown in the ultra-widefield color fundus photograph image (Figure 1D), and the SS-OCT images showed the presence of a posterior vitreous detachment (Figure 1E, 1F). The magnified image (Figure 1G) and a B-scan of SS-OCT (Figure 1H) revealed a hypo-reflective lesion in the choroid beneath the elevation. An image of the widefield enface-OCT (Xephilio OCT-S1; Canon Tokyo, Japan) revealed a large vortex vein ampulla in the posterior pole of her right eye (Figure 2A).

**Figure 3:**
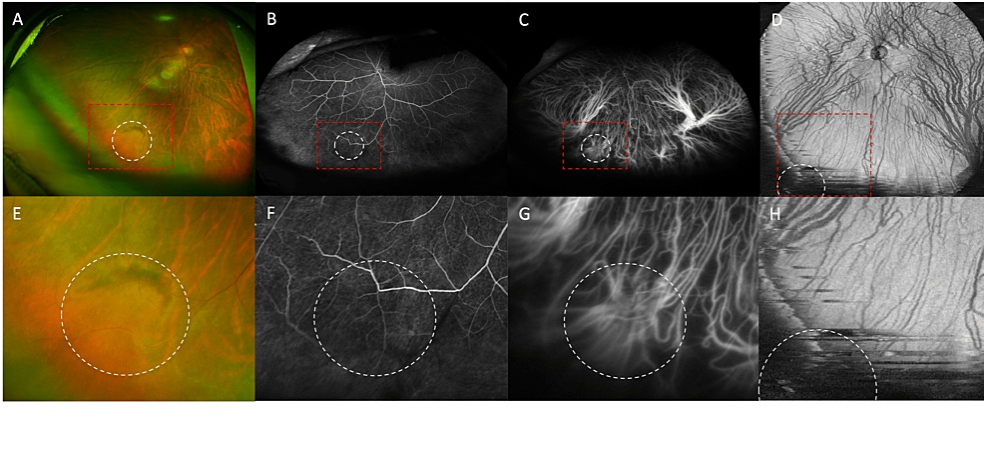
Ultra-widefield color fundus photograph, ultra-widefield fluorescein angiography (UWFA), ultra-widefield indocyanine green fluorescence angiography (UWICGA), and enface-OCT of the left eye with right inferior gaze. The area of the retinochoroidal elevation are surrounded by white round dotted lines. The elevation, which was not obvious in the primary gaze position, is observed in the ultra-widefield color fundus photograph (A, E) with a right inferior gaze. In the UWFA (25 seconds) (B, F), there is no obvious abnormality in the area of the elevation, while in the UWICGA (24 seconds) (C, G), the same area shows a vortex vein ampulla with no congestion of the dye. Enface-OCT of the left eye with a right inferior gaze (D, H) could not identify the elevated lesion due to smaller angle of view. Enlarged images of the red dotted square area in A, B, C, and D are shown in E, F, G, and H, respectively.

Ultra-widefield fluorescein angiography (UWFA) (Figure 2B) and UWICGA (Figure 2C) of her right eye performed with the Optos® California device (Optos, Marlborough, MA USA) showed a comparable result with enface-OCT. Although in the widefield enface-OCT image, the UWFA and UWICGA of her left eye with primary gaze showed no abnormality (Figure 2D-2F), ultra-widefield color fundus photograph, UWFA, UWICGA, and widefield enface-OCT of her left eye with right inferior gaze showed that the elevated lesion was consistent with the vortex vein ampulla (Figure 3A-3H).

Compression of her left eye with Trans Equator® lens (Volk, Mentor, OH, USA) resulted in a diminishing of the ridge, and the release of the ocular compression resulted in an enlargement of the ridge (Video 1). 

**Video 1:** Elevated lesion of the left eye observed with contact lens. A movie and screenshots of the elevated retinochoroidal ridge of the left eye observed with a slit-lamp microscope and the Trans Equator® lens are shown. The retinochoroidal ridge can be observed with no pressure on the eyeball. However, when the eye is compressed with the Trans Equator® lens, the height of the retinochoroidal ridge is decreased, and when the pressure is released, the height is increased again.

These results indicate that the elevation was a varix of vortex vein ampulla. After six months of follow-up, the elevated lesion in her left eye showed no change of appearance.

## Discussion

The imaging of retinochoroidal vessels has advanced dramatically with the development of various imaging modalities. Ultra-widefield imaging devices have contributed to the understanding and elucidation of various pathological conditions by providing a holistic image of the retinochoroidal vasculature in normal and abnormal eyes [5,6,7].

Posterior vortex vein is a condition in which the vortex vein, which is usually seen at the equator, is present in the posterior pole. The posterior vortex veins were classified into five types according to the site of exit from the eye: around the optic nerve, in the macular area, along the border of staphyloma, along the margin of macular atrophy or large peripapillary conus, and elsewhere [1]. The second type is also called macular vortex vein, and our case is of this type [2]. Posterior vortex veins were observed in only one of 42 eyes (2.4%) with a normal refraction within ±3D, while 61 of 255 eyes (23.9%) with a high myopia of -8.25D or more [8]. Furthermore, posterior vortex veins are significantly more common in highly myopic eyes with staphyloma, suggesting an association between them [1]. Previous reports have mainly used ICGA; however, in 2021, the first report of the observation of a posterior vortex vein by OCTA was published, and OCTA was considered to be useful for the detection of posterior vortical veins [9]. Enface-OCT can clearly visualize the choroidal vessels of the posterior pole [10] and show a greater distinctness than en-face OCTA imaging [11]. Thus, in this study, we applied enface-OCT to confirm a well-defined macular vortex vein.

Clinically, the varix of vortex vein ampulla may appear as an elevated mass, which is ill-defined and dark red in color [3]. The cite, number, and thickness of the varix vary for each patient [4]. Although a varix of the vortex vein ampulla does not pose a direct threat to vision and does not require treatment, it might be a risk factor for the occurrence of suprachoroidal hemorrhage in high myopia [12]. It is clinically important to consider the differential diagnosis of chorioretinal mass lesions, such as choroidal melanoma, choroidal nevus, choroidal secondaries such as metastases to the choroid, subretinal hemorrhage, or choroidal hemangioma [4]. ﻿Varix of the vortex vein ampulla usually becomes more prominent with positioning of the eyes in the direction of the lesion [13]. However, it flattens or collapses in other fields of gaze or when slight pressure is applied to the globe [14]. ﻿The dynamic or fluctuating nature of the lesion is the most helpful clue in the diagnosis [13]. A previous report indicates that UWICGA is a beneficial imaging modality for the identification and characterization of vortex vein ampullae [5]. In the present case, UWICGA of the right inferior gaze of the left eye showed a vortex vein ampulla in an area corresponding to the varix of vortex vein ampulla observed by the ultra-widefield color fundus photograph, although no abnormal findings such as congestion of the dye were observed. The same area could not be visualized by enface-OCT due to the smaller angle of view. The development of OCT and OCTA with a wider angle of view is expected to improve the non-invasive detection and diagnostic accuracy of varix of vortex ampulla in the future.

## Conclusions

The present case showed two different vortex vein anomalies, macular vortex vein in the right eye and varix of vortex ampulla in the left eye. As far as we know, this is the first report of such different vortex vein anomalies coexisting in a single patient. Multimodal imaging is useful for visualizing choroidal vascular anomalies.
